# GPR52 regulates cAMP in T cells but is dispensable for encephalitogenic responses

**DOI:** 10.3389/fimmu.2022.1113348

**Published:** 2023-01-24

**Authors:** Paula F. Krieg, Jana K. Sonner, Roberta Kurelic, Jan Broder Engler, Marlena F. Scharenberg, Simone Bauer, Viacheslav O. Nikolaev, Manuel A. Friese

**Affiliations:** ^1^ Institute of Neuroimmunology and Multiple Sclerosis, Center for Molecular Neurobiology Hamburg, University Medical Center Hamburg-Eppendorf, Hamburg, Germany; ^2^ Institute of Experimental Cardiovascular Research, University Medical Center Hamburg-Eppendorf, Hamburg, Germany

**Keywords:** cAMP, experimental autoimmune encephalomyelitis, FRET, GPCR, multiple sclerosis, T cells

## Abstract

G-protein coupled receptors (GPCR) regulate 3’,5’-cyclic adenosine monophosphate (cAMP) levels in T cells. cAMP as ubiquitous second messenger is crucial for adequate physiology of T cells by mediating effector T cell (Teff) function as well as regulatory T cell (Treg)-mediated immunosuppression. Several GPCRs have been identified to be crucial for Teff and Treg function. However, the role of the orphan, constitutively active Gs-coupled GPCR GPR52 is unknown. Here we show that GPR52 regulates cAMP levels in T cells but does not affect T cell function. We found that stimulation of transfected HEK cells or primary T cells with a GPR52 agonist results in a rise of intracellular cAMP. However, neither *Gpr52* deficiency nor pharmacological modulation of GPR52 by antagonists or agonists affected T cell activation, differentiation, and proliferation or Treg-mediated immunosuppression. Moreover, *Gpr52* deletion did not modify the clinical disease course of experimental autoimmune encephalomyelitis (EAE). Our results demonstrate that a modulation of cAMP levels in T cells does not inevitably result in altered T cell function. While we could not identify an obvious role of GPR52 in *in vitro* T cell assays and *in vivo* CNS autoimmunity, it might regulate T cell function in a different context or affect the function of other GPR52-expressing cells.

## Introduction

G-protein coupled receptors (GPCR) are a class of seven-transmembrane domain proteins that can be activated by an array of different ligands, including peptides, hormones, proteins, or ions making them a target of numerous pharmaceuticals (1, 2). A well-described pathway regulated by GPCRs mediates the generation of the ubiquitous second messenger cyclic 3′,5′-adenosine monophosphate (cAMP). Gs-coupled GPCRs can either be activated by an extracellular ligand or exert a constitutive activity resulting in the subsequent activation of adenylyl cyclases (AC). Upon activation, AC can catalyze the formation of cAMP from ATP. Phosphodiesterases (PDE) on the other hand can hydrolyze cAMP, resulting in a decrease in intracellular cAMP levels (3, 4).

In the adaptive immune system, cAMP acts as an important regulator of T cell function. In effector T cells (Teff), modulation of intracellular cAMP concentrations alters downstream pathways. Increasing cAMP levels result in the inhibition of T cell activation and proliferation (5, 6). Rising levels of cAMP activate protein kinase A (PKA) (7), which subsequently suppresses the expression of genes that are associated with T cell activation and proliferation *via* several transcription factors, such as CREB, NFAT and NF-κB (3, 8).

Furthermore, regulatory T cells (Treg) are characterized by elevated basal levels of cAMP compared to Teffs. High cAMP concentrations in Tregs are partly due to forkhead box P3 (FOXP3) expression, a master transcriptional regulator of Tregs. FOXP3 leads to an inhibition of PDE3B, while at the same time an inhibition of miR-142-3p results in increased activation of AC9. These FOXP3-mediated mechanisms ensure high levels of intracellular cAMP in Tregs. In Teffs, FOXP3 is absent resulting in low cAMP levels under basal conditions (9, 10). The discrepancy of cAMP levels in Tregs and Teffs is important for Treg-mediated immunosuppression, which is essential to maintain homeostasis and immunological self-tolerance (11, 12). Upon cell-cell contact, Tregs have been shown to transfer cAMP *via* gap junctions into Teffs, resulting in increased cAMP in target Teffs (13). Furthermore, ectonucleotidases CD39 and CD73 are highly expressed on Tregs. These enzymes degrade extracellular ATP to adenosine, which binds to the adenosine receptor A_2A_R on Teffs. Activation of this transmembrane receptor results in AC activation leading to an increased synthesis of intracellular cAMP levels (14, 15).

An imbalance of intracellular cAMP in T cells can lead to auto-aggressive Teffs or a disruption of the immunosuppressive capacity of Tregs which might result in autoimmune diseases, such as multiple sclerosis (MS). In MS, autoreactive T cells and other immune cells infiltrate the central nervous system (CNS), leading to an inflammation that results in oligodendrocyte damage leading to demyelination and neurodegeneration (16–19). Exuberant activation of Teffs has been identified in MS as a possible driver of disease onset and progression (20, 21). On the other hand decreased Treg numbers and/or their impaired ability to suppress autoreactive immune cells has been proposed to be accountable for autoimmune diseases (22). It has been demonstrated that Tregs are capable of controlling the severity of experimental autoimmune encephalomyelitis (EAE), the animal model of MS. Reduced Treg function exerted by antibody-mediated depletion results in exacerbated severity (23), while adoptive transfer of Tregs rescues severe EAE disease courses (24, 25). In line with that, Tregs derived from blood samples of MS patients appear to have a decreased suppressor function compared to Tregs from healthy controls (26).

However, which orphan GPCRs expressed by T cell subsets are involved in the generation of intracellular cAMP levels and therefore modify T cell function, remains unknown. In this study, we compiled GPCRs expressed in Tregs and Teffs. We investigated the role of the orphan, constitutively active Gs-coupled GPR52 in regulating cAMP levels in T cells and addressed its potential as a therapeutic target for the treatment of autoimmune diseases. In *in vitro* studies, we could show that pharmacological manipulation of GPR52 alters intracellular cAMP levels in T cells. However, *Gpr52* deletion or pharmacological activation did not alter T cell function. Moreover, genetic deletion of *Gpr52* did not modulate the EAE disease course, which has led us to conclude that GPR52 is important for cAMP modulation, but dispensable for T cell function and Treg-mediated immunosuppression.

## Results

### GPR52 regulates cAMP levels in T cells

**Figure 1:**
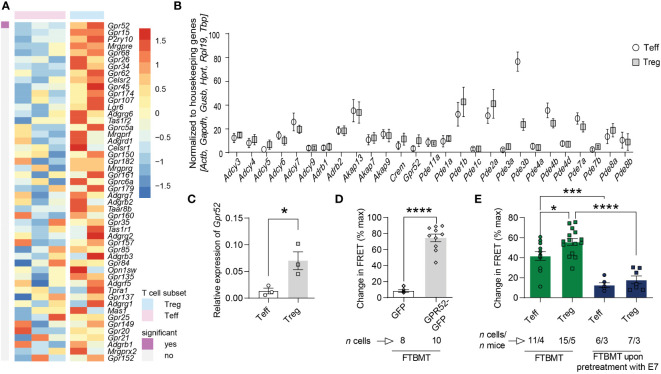
cAMP-relevant gene expression and cAMP changes upon GPR52 pharmacological interventions. **(A)** Expression of orphan GPCRs in CD4^+^Foxp3^–^ Teffs and CD4^+^Foxp3^+^ Tregs was analyzed using available RNA sequencing data (Heng and Painter, 2008). Shown are top 50 genes ranked as highest Treg-biased expression. Treg: *n* = 2; Teffs *n* = 3. **(B)** NanoString nCounter analysis of cAMP-relevant genes in Treg to Teff, and **(C)** RT-qPCR expression of *Gpr52* in T cell subsets. NanoString: *n* = 6 mice per group; RT-qPCR: *n* = 3 mice per group. For analysis of NanoString nCounter data, Wilcoxon matched pairs signed rank test identified differential expression of *Gpr52, Pde1b, Pde2a, Pde3b, Pde4b, Pde7a*, and *Pde7b* on individual level, but *P*_adjust_ was > 0.05 after Benjamini Hochberg correction. **(D)** Quantification of maximal FRET changes in transfected HEK293A cells is depicted as percentage of FRET response upon addition of 0.5 µM FTBMT in comparison to maximal FRET response generated by addition of 10 µM FSK and 100 µM IBMX (3-isobutyl-1-methylxanthine). n of cells is indicated. **(E)** Quantification of FRET changes (as in D) upon FTBMT (500 nM) treatment of non-activated T cell subsets with and without GPR52 antagonist (E7, 10 µM) pre-treatment. n of cells and mice are indicated. Statistical analyses were performed using limma, followed by Benjamini-Hochberg correction with *P* < 0.05 for significant finding **(A)**, Wilcoxon matched pairs signed rank test followed by Benjamini-Hochberg correction **(B)**, paired Student’s *t*-test **(C)** or unpaired Student’s *t*-test **(D)**, and one-way ANOVA followed by Sidak multiple comparison tests **(E)**. *P < 0.05, ***P < 0.001 and ****P < 0.0001.

To identify orphan GPCR engaged in the generation of cAMP in mouse T cells, we analyzed available RNA sequencing data (27) and found a subset-specific expression of several orphan GPCRs (**Figure 1A**; **Figures S1**, **S2**). The orphan Gs-coupled GPR52 that has previously been shown to alter intracellular cAMP levels in transfected HEK cells and primary neurons (28, 29) was also expressed in T cells and differentially higher in Tregs compared to Teffs. Since GPR52 has not been studied in the context of T cell function, we selected this GPCR for further functional investigations. We verified the differential expression of GPR52 in mouse T cell subsets using multiplexed gene expression analysis and RT-qPCR (**Figures 1B, C**; **Figure S3**). Next, we tested the specificity of 4-(3-(3-fluoro-5-(trifluoromethyl)benzyl)-5-methyl-1H-1,2,4-triazol-1-yl)-2-methyl-benzamide (FTBMT), a selective GPR52 agonist, in HEK293A cells. As GPR52 is not endogenously expressed in HEK293A cells, we transfected them with the cytosolic FRET-based cAMP sensor *Epac1-camps* together with *Gpr52-GFP* or *GFP*-control. We observed a significant increase in cAMP levels upon FTBMT treatment of *Gpr52*-transfected HEK293A cells in comparison to control-transfected cells (**Figure 1D**; **Figures S4A, B**). Having validated FTBMT specificity, we detected, in line with expression data, higher cAMP production in Tregs compared to Teffs from *CAG-Epac1-camps* transgenic mice (30) after FTBMT pre-treatment (**Figure 1E**; **Figures S4C, D**). According to the calibration curve of our cAMP biosensor (31) in its relevant range, the difference of approx. 15% response corresponds to 1.5–2 µM higher intracellular cAMP in Treg. By contrast, pre-treatment of T cell subsets with E7, an antagonist of GPR52 (28), decreased intracellular cAMP levels in both Teffs and Tregs compared to FTBMT treatment alone and resulted in a comparable FRET signal in Teffs and Tregs (**Figure 1E**; **Figures S4E, F**). Together, this indicates that GPR52 activation on T cells increases their intracellular cAMP levels with Tregs mounting a stronger response to the selective GPR52 agonist than Teffs.

### *Gpr52* deficiency does not alter T cell function

**Figure 2:**
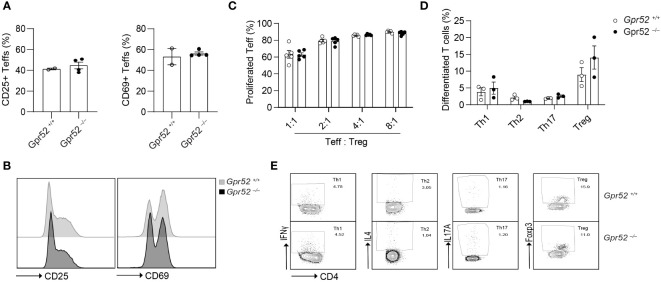
Gpr52 deficiency does not alter T cell function. **(A)** Teffs activation of cells derived from *Gpr52^–/–^* (*n* = 3) or *Gpr52^+/+^* (*n* = 4) mice. **(B)** Representative FACS blots of CD69 and CD25 expression in *Gpr52^–/–^* and *Gpr52^+/+^*. **(C)** *Gpr52^–/–^* and *Gpr52^+/+^* Treg-mediated suppression on wildtype Teffs proliferation; *n* = 5 mice per group. **(D)** T cell differentiation from naïve CD4^+^ T cells to Th1, Th2, Th17 or Tregs of *Gpr52*-deficient and -proficient T cells. *Gpr52^–/–^ n* = 3 mice; *Gpr52^+/+^ n* = 3 mice. **(E)** Representative FACS blots of differentiated Th1, Th2, Th17 and Treg cells from naïve CD4+ T cells derived from *Gpr52^–/–^* and *Gpr52^+/+^* mice. Statistical analyses were performed using unpaired Student’s t-test (CD25) or Mann-Whitney U test (CD69) **(A)**, and multiple t-tests followed by Benjamini-Hochberg correction **(B, C)**.

As we have shown that pharmacological manipulation of GPR52 alters intracellular cAMP levels, we hypothesized that T cell function is also affected by *Gpr52* deletion. To test this, we used primary T cells isolated from *Gpr52*-deficient mice with a verified knockout of *Gpr52* as shown by RT-qPCR (**Figure S5**). However, *Gpr52* deficiency did not result in an altered T cell activation by antibody-mediated CD3 and CD28 stimulation (**Figures 2A, B**). Moreover, *Gpr52* deletion did not affect the immunosuppressive capacity of Tregs (**Figure 2C**; **Figure S6**). Differentiation of naïve CD4+ T cells into T helper subsets (Th1, Th2, Th17, and Treg) was also not different in T cells derived from *Gpr52-*deficient mice compared to wildtype littermate controls (**Figures 2D, E**). Thus, GPR52 is dispensable for T cell activation, differentiation and Treg-mediated immunosuppression in *in vitro* assays.

### T cell function is unaltered by GPR52 agonists or antagonists

**Figure 3:**
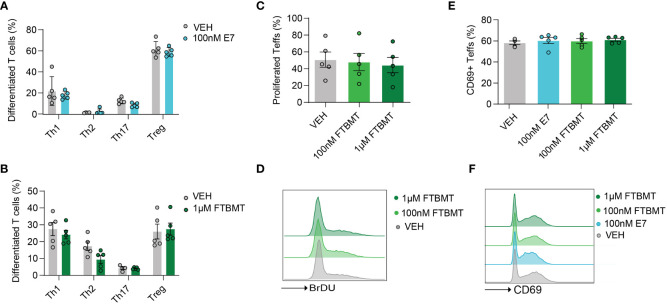
Effects of E7 and FTBMT treatment on *in vitro* T cell function. **(A, B)** Differentiation of naïve CD4^+^ T cells to Th1, Th2, Th17 and Tregs after E7 **(A)** and FTBMT **(B)** treatment. E7 *n* = 5 mice; FTBMT *n* = 5 mice. **(C)** T cell proliferation upon FTBMT treatment; *n* = 5 mice. **(D)** Representative FACS blot for T cell proliferation upon FTBMT treatment. **(E)** Effect of E7 treatment on early marker of T cell activation CD69; *n* = 5 mice. **(F)** Representative FACS blot depicting the expression of early activation marker upon E7 or FTBMT treatment. Statistical analyses were performed using multiple paired *t*-tests **(A, B)** or multiple paired Mann-Whitney U test **(E)** followed by Benjamini-Hochberg correction and one-way ANOVA followed by Dunnett’s correction **(C, D)**.

As *Gpr52* deficiency had no apparent impact on T cell function, we next determined whether treatment of T cells with the selective GPR52 antagonist E7 (28) or agonist FTBMT (32) results in altered T cell function. We first tested the effect of E7 and FTBMT treatment on wildtype T cells derived from C57BL/6. Treatment with 100 nM E7 did not alter the differentiation of naïve CD4+ T cells to Th1, Th2, Th17 or Tregs (**Figure 3A**; **Figure S7A**). Similarly, FTBMT agonist treatment did not alter differentiation from naïve CD4+ T cell into T cell subsets (**Figure 3B**; **Figure S7B**). FTBMT did neither affect T cell proliferation (**Figures 3C, D**) nor did FTBMT or E7 alter T cell activation (**Figures 3E, F**) of CD4+ T cells. Upon higher concentration of E7, as suggested in literature (28, 29), increased cell death was observed (**Figure S7C, D**). Toxic effect of E7 was observed on both, *Gpr52-*deficient and wildtype T cells (**Figure S7E**). In line with that, decrease of T cell activity upon higher concentration of E7 was observed in both, *Gpr52*-proficient and *Gpr52*-deficient T cells, independent of GPR52 abundance (**Figure S7F, G**). Together, the GPR52 agonist FTBMT did not alter T cell function while the observed effects after E7 antagonist treatment at high concentrations were GPR52-independent.

### *Gpr52* deficiency does not impact on EAE disease course

**Figure 4:**
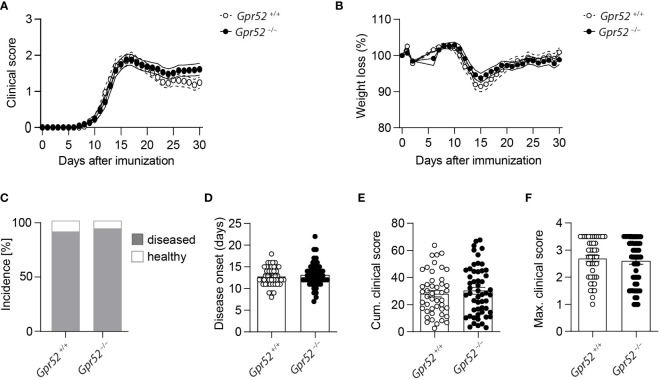
*Gpr52* deficiency does not alter EAE severity. **(A, B)** Clinical score **(A)** and relative weight **(B)** loss of *Gpr52^–/–^* and *Gpr52^+/+^*. **(C)** Incidence of diseased mice. **(D–F)** Disease onset **(D)**, cumulative clinical score **(E)**, maximal clinical score **(F)** reached during the EAE. Disease onset was defined as first day at which animals showed clinical symptoms. *Gpr52^–/–^ n* = 53 mice; *Gpr52^+/+^ n* = 46 mice. Statistical analyses were performed using AUC followed by Student’s t-test **(A, B)**, Fishers Exact test **(C)**, Mann-Whitney U test **(D, F)**, and Student’s *t*-test **(E)**.

As cAMP is crucial for Teff functions and the immunosuppressive capacity of Tregs, we finally tested whether GPR52 exerts its function in an autoimmune response. To do so, we induced three independent EAE in *Gpr52*-deficient mice and age- and sex-matched wildtype littermate controls. However, neither clinical score (**Figure 4A**), nor weight loss differed between the experimental groups (**Figure 4B**). Also the EAE incidence (**Figure 4C**), disease onset (**Figure 4D**), defined as day when first symptoms were recorded, cumulative clinical score (**Figure 4E**) and maximal clinical score (**Figure 4F**) did not differ between *Gpr52*-deficient animals and wildtype littermate controls. Thus, we compiled evidence that GPR52 is dispensable for encephalitogenic T cell generation and CNS auto-aggression.

## Discussion

GPCRs are important modulators of intracellular cAMP levels. In T cells, the tight regulation of intracellular cAMP is crucial in controlling adaptive immune responses. Activation of Gs-coupled GPCRs results in a local rise of cAMP in intracellular nanodomains (33). Rising cAMP concentration activates several downstream pathways, starting with the modulation of PKA, guanine-nucleotide-exchange factor (GEF) exchange proteins (Epacs), and cyclic nucleotide-gated channels (34, 35). The PKA-mediated pathway, including modification of several transcription factors, such as CREB and NFAT, as well as downstream kinase activation, like Lck and Csk, is responsible for cAMP-mediated alterations in Teff responses (36, 37). Moreover, cAMP is a key factor of Treg-mediated suppression of Teffs and other immune cells (38, 39). Thus, GPCRs are promising pharmaceutical targets to modulate T cell function and autoimmune diseases. Several GPCRs have been considered in this context, among them the PGE receptor (40, 41), chemokine receptors (42), and other orphan GPCRs (43, 44). However, the function of several T cell-expressed orphan GPCRs remains unknown.

By analyzing the expression of orphan and de-orphanized GPCRs expressed in Teffs vs. Tregs, we detected differentially expressed GPCRs. Since the orphan GPR52 has not been studied in the adaptive immune system and we were able to validate higher transcript levels in Tregs compared to Teffs, we decided to further investigate this promising candidate. The constitutively active GPR52 is a Gs-coupled GPCR and hence involved in the cAMP-generating pathway (32, 45). Pharmacological inhibition of GPR52 with the antagonist E7 or activation with the agonist FTBMT has been reported to modulate intracellular cAMP levels in transfected HEK cells and primary neurons (28, 32). Furthermore, an involvement of GPR52 in Huntington’s disease has been described. A global *Gpr52* knockout, as well as GPR52 antagonist (E7) treatment has been reported to reduce Huntington’s disease-related symptoms and mutant huntingtin (mHTT) levels in mouse primary striatal neurons (28, 29).

In this study, we could verify the Gs-coupled activity of GPR52 by FRET imaging of transfected HEK cells (46) and furthermore prove that GPR52 alters cAMP levels in T cells. In accordance with increased expression of *Gpr52* in Tregs compared to Teffs, stimulation of GPR52 differentially altered cAMP levels in Tregs and Teffs. However, in *in vitro* assays for T cell function, we could show that neither *Gpr52* deficiency, nor pharmacological manipulation affect T cell function. Similarly, deletion of *Gpr52* did not alter the severity of EAE in any of the assessed parameters. Thus, even though we demonstrated the regulation of intracellular cAMP *via* GPR52 in T cells, we could not detect a functional effect of pharmacological manipulation or genetic deletion of *Gpr52* on T cell physiology *in vitro* and in the context of EAE.

Although we could show that GPR52 manipulation alters cAMP levels in T cells, this cAMP rise likely occurs in GPR52-specific nanodomains, so-called receptor-associated intracellular nanodomains (RAIN) (33, 47). These specific nanodomains differentially modulate downstream signaling. However, the rise of cAMP in GPR52-specific RAINs seems not to impact T cell physiology in the assays and disease model that we tested in our experimental setup. Moreover, other well-described key regulators of intracellular cAMP in Treg, such as PDE3B, could be strong modulators that overrule the manipulation of a single Gs-coupled GPCR and the subsequent rise in cAMP in associated RAINs. Whether the GPR52-associated RAIN is an important modulator of appropriate functions in other cells, e.g. in neurons (28) or other cells of the immune system remains an open question and requires further in depths analysis. Furthermore, we could show that the previously described effects of the GPR52 antagonist E7 (28) were not mediated by GPR52 in our assays but rather resulted from toxic effects of E7 on T cells. Whether that is a T cell-specific effect or also applicable to other cell types is currently unclear but should be considered when using this compound.

Together, we could show that GPR52 is differentially expressed in T cell subsets. Furthermore, we proved that GPR52 is accountable for increasing levels of cAMP in T cells upon activation with FTBMT with a higher cAMP elevation in Tregs compared to Teffs. We could also show that neither GPR52 stimulation with FTBMT, nor *Gpr52* deficiency altered T cell function in any of the assessed *in vitro* assays. Besides that, we observed a toxic effect of the published agonist E7 on T cells. In the *in vivo* EAE mouse model we could show that *Gpr52* deficiency does not affect the clinical severity of the disease course. Therefore, we conclude that GPR52 modulates cAMP in T cells but its functional impact has yet to be discovered.

## Material and methods

### Animals

Seven- to 12-week-old C57BL/6 wildtype, CAG-Epac1-camps and *Gpr52*-deficient mice (Gpr52^tm1Kohi^) were used in this study (30, 46, 48). *Gpr52*-deficient mice were kindly provided by Takeda Pharmaceutical Company Limited. Mice were kept under specific pathogen-free conditions in the central animal facility of the University Medical Center Hamburg-Eppendorf (UKE) and were handled in accordance to international and national animal welfare guidelines with the organ isolation procedures approved by the *Behörde für Justiz und Verbraucherschutz* Hamburg (ORG1010, ORG946 and ORG1075). Induction of experimental autoimmune encephalomyelitis and tissue sample collection was licensed under No. 45/17 and 83/19.

### HEK293A transfection and FTBMT treatment

HEK293A cells were cultured in Dulbecco’s modified Eagle’s medium (DMEM, Sigma Aldrich) at 37°C and 5% CO_2_. HEK293A were plated on 25 mm glass coverslips. To ensure proper cell attachment, cells were cultured overnight and transfected with a mixture consisting of plasmid DNA (0.5 μg/well), non-supplemented DMEM, and Lipofectamine 2000 (Thermo Fisher Scientific, following the manufacturer’s transfection protocol) in a total volume of 50 μl/well. Cells were transfected with cytosolic FRET-based sensor, Epac1-camps, and pcDNA3 (empty vector) or GPR52-encoding plasmid labeled with GFP. 40–48 hours post-transfection, cells with proper expression of the plasmid DNA were used to validate GPR52 agonist drug (FTBMT) specificity *via* real-time FRET measurements described below.

### Förster resonance energy transfer imaging

For live-cell imaging *via* FRET, spleens from eight- to 12-week-old CAG-Epac1-camps transgenic mice (30) were collected on ice-cold PBS. CD4+ T cells were obtained from single-cell suspension by negative selection following the manufacturer’s protocol of murine CD4+ T cell Isolation Kit (Miltenyi Biotec). Separation of CD4+CD25- and CD4+CD25+ T cells were processed by further labeling with CD25-biotin antibody (Miltenyi Biotec). T cell activation was achieved upon overnight culture of T cell subsets in presence of Dynabeads Mouse T-Cell Activator CD3/CD28 (Thermo Fisher) at 37°C and 5% CO_2_. Real-time measurements were performed on T cells plated on 25 mm glass coverslips coated with poly-D-Lysine (Sigma Aldrich). For glass coverslips, an Autofluor cell chamber was used, as well as FRET buffer (144 mM NaCl, 5.4 mM KCl, 1 mM MgCl_2_, 1 mM CaCl_2_, 10 mM HEPES; pH 7.3), to dilute chemical compounds tested. Non-adherent cells were washed before the start of the measurement. To monitor FRET response Leica DMI 3000 B was used, an inverted fluorescent microscope equipped with an oil-immersion 63x/1.40 objective and a MicroManager 1.4. software. As the fluorescent light source CoolLED was used, to excite cyan fluorescent protein (CFP) at 440 nm. Beam-splitter, DV2 Dual View (Photometrics; Cube 05-EM, 505 dcxr, D480/30, D535/40), was used to split the emission light into CFP and yellow fluorescent protein (YFP) channel, simultaneously monitored on a CMOS (OptiMOS, QImaging) camera chip. Images were taken every 5- or 10-seconds. Data were analyzed by ImageJ (RRID: SCR:003070) and Microsoft Excel for offline corrections with a spectral bleed-through correction factor as described (31).

### NanoString nCounter analysis

Lymph nodes (superficial cervical, axillary, brachial, inguinal) and spleen from C57BL/6 wildtype mice were collected in ice-cold PBS. CD4+ T cells were enriched by negative selection using the MojoSort CD4+ T cell isolation kit (BioLegend). CD4+ T cells were stained for T cell surface marker for 30 min at 4°C. Antibodies that were used are listed in **Supplementary Table 1**. Dead cells were excluded by adding Alexa Fluor 750 NHS Ester (Succinimidyl Ester) (Thermo Fisher). Nonspecific Fc receptor-mediated antibody binding was minimized by blocking with TruStain FCX anti-mouse CD16/32 (clone 93, BioLegend). Stained cells were FACS-purified using a FACS Aria III cell sorter (BD Bioscience). RNA of sorted samples was isolated using RNeasy Mini Kit (Qiagen). The yield of total RNA was determined by using the Qubit RNA high sensitivity, broad range, and extended range Assay Kit (Thermo Fisher Scientific). mRNA expression was measured with the NanoString nCounter FLEX Analysis System (RRID: SCR_021712, NanoString Technologies) using 30–35 ng of total RNA. The custom-made CodeSet for 36 genes including 6 housekeeping genes (*Actb, Gapdh, Gusb, Hprt, Rpl19, Tbp*) was hybridized to total RNA for 16 hours at 67°C. The expression data were analyzed utilizing the NanoString nSolver Analysis Software 4.0. Raw data were analyzed and normalized to the housekeeping genes together with quality control performed using nSolver 4.0 User Manual in addition to default settings and algorithm within the nSolver Analysis software.

### T cell activation assay

For T cell activation assay, lymph nodes (superficial cervical, axillary, brachial, inguinal) and spleen from C57BL/6 wild-type mice or *Gpr52* ^–/–^ and respective littermate controls were collected in ice-cold PBS. CD4+ T cells were isolated from single-cell suspension using the MojoSort CD4+ T cell isolation kit (BioLegend) according to the manufacturer’s protocol. CD4+ T cells were seeded in an anti-CD3 (1 µg/ml; clone 145-2CL11, BioLegend)-coated 96-well plate. Cells were supplemented with compounds (E7 and FTBMT) and soluble anti-CD28 (2 µg/ml; clone 37.51, BioLegend). Samples were incubated for 6 hours at 37°C and 5% CO_2_. After that cells were stained for T cell surface markers for 30 minutes at 4°C. Dead cells were excluded by adding Alexa Fluor 750 NHS Ester (Succinimidyl Ester) (Thermo Fisher). Nonspecific Fc receptor-mediated antibody binding was minimized by blocking with TruStain FCX anti-mouse CD16/32 (clone 93, BioLegend). After fixation and permeabilization at RT with eBioscience™ Foxp3/Transcription Factor Staining Buffer Set (Thermo Fisher), FOXP3 was stained intranuclearly for 30 min at RT. Cells were stained with antibodies listed in **Supplementary Table 1**. Samples were acquired on the BD FACS LSR II analyzer (BD Bioscience) or FACSymphony A3 (BD Bioscience). Data were analyzed using FlowJo (version 10, BD Bioscience, RRID: SCR_008520).

### T cell proliferation assay

For T cell proliferation assay lymph nodes (superficial cervical, axillary, brachial, inguinal) and spleen from C57BL/6 wildtype mice were collected in ice-cold PBS. Single-cell suspension was generated and splenocytes were seeded in an anti-CD3 (clone 145-2CL11, BioLegend)-coated 96-well plate. Cells were supplemented with soluble anti-CD28, IL2 (Peprotech) and FTBMT (100 nM and 1 µM; MedChemExpress). Samples were incubated for 72 hours at 37°C and 5% CO_2_. During the last 16 hours cells were pulsed with 1 µg/ml bromodeoxyuridine (BrdU, BioLegend). Cells were stained for surface markers, fixated and permeabilized as described above. Next, cells were incubated in PBS supplemented with Ca^2+^ and Mg^2+^ supplemented with 40 KU/ml DNase I (Merck) for 1 hour at 37°C and 5% CO_2_. After DNA digestion, Foxp3 and incorporated BrdU was stained for 30 min at RT. Used antibodies are listed in **Supplementary Table 1**. Samples were acquired and data were analyzed as indicated above.

### T cell differentiation assay

For T cell differentiation assays lymph nodes (superficial cervical, axillary, brachial, inguinal) and spleen from C57BL/6 wildtype mice or *Gpr52* ^–/–^ and littermate controls were collected in ice-cold PBS. Naïve CD4+ T cells were isolated from single-cell suspension using the MojoSort naïve CD4+ T cell isolation kit (BioLegend) according to manufacturer’s protocol. Naïve CD4+ T cells were seeded in an anti-CD3 (clone 145-2CL11, BioLegend)-coated 96-well plate and supplemented with soluble anti-CD28, compounds (100 nM E7, 1 µM E7 or 1 µM FTBMT), as well as respective cytokines and antibodies required for induction of each T cell subset (see **Supplementary Table 2**). Cells were incubated for 72 hours at 37°C and 5% CO_2_. During the last 5 hours of incubation, cells were stimulated with 1 µg/ml ionomycin (Sigma Aldrich), 20 ng/ml phorbol 12-myristate 13-acetate (PMA, Sigma Aldrich), and brefeldin A (BioLegend) to induce cytokine production and accumulation. Cells were stained for surface markers, fixed and permeabilized as well as stained for intracellular marker as indicated above. Antibodies used are listed in **Supplementary Table 1**. Samples were acquired and data were analyzed as described above.

### Treg suppression assay

For Treg suppression assay, lymph nodes (superficial cervical, axillary, brachial, inguinal) and spleen from *Gpr52* ^–/–^ and littermate controls were collected in ice-cold PBS. Teffs and Tregs were isolated from single-cell suspension using CD4+ CD25+ Regulatory T cell isolation kit (Miltenyi Biotec) according to manufacturer’s protocol. Staining of wildtype Teffs was performed with CellTrace™ CFSE Cell Proliferation Kit (Thermo Fisher). Briefly, Teffs were stained for 15 minutes at 37°C with reconstituted CFSE in PBS containing 0.1% BSA (Miltenyi Biotec). Tregs and labeled wildtype Teffs were seeded in 96-well plate together with Dynabeads™ mouse T-activator CD3/CD28 (ratio Teffs:beads 1:1; Thermo Fisher) and IL-2 (10 IU/ml, Peprotech). Cells were incubated for 72 hours at 37°C and 5% CO_2_. Cells were stained for surface markers, fixed, permeabilized, and stained for intranuclear FOXP3 as indicated above. Antibodies used are listed in **Supplementary Table 1**. Samples were acquired and data were analyzed as indicated above.

### Cell viability assay

T cells were isolated from lymph nodes (superficial cervical, axillary, brachial, inguinal) and spleen from C57BL/6 wild-type mice from single-cell suspension using the MojoSort CD4+ T cell isolation kit (BioLegend) according to the manufacturer’s protocol. After, CD4+ T cells were supplemented with compounds (E7 and FTBMT) at increasing doses and incubated for 72 hours at 37°C and 5% CO2. Cell viability was assessed using the CellTiter-Glo Luminescent Cell Viability Assay (Promega) on a Spark 10M multimode microplate reader (Tecan).

### GPR52 expression analysis in *Gpr52*-deficient mice

Teffs and Tregs of seven- to ten-week-old *Gpr52*-deficient mice and littermate controls were isolated from peripheral lymphatic organs (axillary, brachial, superficial, cervical, inguinal lymph nodes, and spleen). T cells were isolated using the MojoSort CD4+ T cell isolation kit (BioLegend) according to the manufacturer’s instructions. Cells were stained with antibodies as indicated in **Supplementary Table 1**. Teffs and Tregs were sorted with FACS Aria III cell sorter (BD Bioscience). Samples were further analyzed using quantitative real-time PCR analysis as described below.

### Experimental autoimmune encephalomyelitis

For induction of EAE, eight- to 12-week-old male and female Gpr52–/– and littermate controls were immunized subcutaneously with 100 µg MOG_35-55_ (peptides and elephants) in complete Freund’s adjuvants (BD Difco) containing 4 mg/ml Mycobacterium tuberculosis (BD Difco). 250–300 ng pertussis toxin (Merck Millipore) was injected intraperitoneally in 100 µl PBS on the day of immunization and 48 hours later. Weight and clinical signs of disease were scored daily from day 7 to day 30. Mice were scored blinded for clinical signs by the following system: 0: no clinical deficits; 1: tail weakness; 2: hind limb paresis; 3: partial hind limb paralysis; 3.5: full hind limb paralysis; 4: full hind and forelimb paresis. Animals that reached a score of 4, or 3.5 for more than 3 days, or lost weight of ≥ 25% of starting weight were euthanized according to the regulations of the local Animal Welfare Act. Graphs show pooled data from 3 independent experiments.

### Quantitative real-time PCR analysis

Total RNA was isolated from MACS-purified Treg and Teff with RNeasy Plus Micro Kit according to manufacturer′s protocol (Qiagen). iScript cDNA Synthesis Kit (Bio-Rad) was used as the manufacturer′s instructions indicated. Primer pairs were synthesized by Eurofins, and the following primer pairs were used: mouse *Gpr52* (forward: 5′-TTGTCTTGCTGACATTTCTGATCA-3′ and reverse: 5′-GGAGCACAGTGAAAGACAAAGATG-3′), and mouse *Tbp* as housekeeping gene (forward: 5′-GTAGCGGTGGCGGGTATC-3′ and reverse: 5′-CATGAAATAGTGATGCTGGGA-3′). To perform quantitive real-time PCR SyberGreen (Perfecta, QuantaBio) was utilized. Samples were analyzed with Rotor Gene-Q cycler and corresponding program for analysis (Qiagen). To obtain relative gene expression level for *Gpr52* ΔCt quantification was employed.

### Statistics

Data shown in bar graphs are shown as mean ± standard error of the mean (SEM). For EAE and functional T cell assays, *n* represents the number of mice. For FRET measurements, number of mice (*n*) and number of single measured cells (*n*) are indicated below the graphs. Number of mice was used for statistical analysis. For heatmap of analysis of available RNA sequencing data (27), differences in gene expression between Teffs and Tregs were tested using limma (49) and genes with a Benjamini-Hochberg corrected *P* value < 0.05 were considered differentially expressed. Statistical analysis of other data was performed using GraphPad software (RRID: SCR_002798). Normal distribution was tested using Kolmogorov-Smirnov or Shapiro-Wilk test. Differences were tested using one-way ANOVA (followed by Dunett’s or Sidak multiple comparison tests), Wilcoxon matched pairs signed rank test, multiple T tests and Mann-Whitney U test (corrected by Benjamini-Hochberg Correction), Area-under-Curve, Fisher’s exact test, Student’s t-test, or Mann-Whitney U test, as appropriate and indicated in the figure legends. Significant differences are indicated as *P < 0.05, **P < 0.01, ***P < 0.001 and ****P < 0.0001.

## Data availability statement

The data discussed in this publication have been deposited in NCBI’s Gene Expression Omnibus (50) and are accessible through GEO Series accession number GPL32955 - NanoString nCounter Mouse T Cell Subset Delineation Panel and GSE221135 - Gene expression of cAMP-relevant genes in T cell subsets.

## Ethics statement

The animal study was reviewed and approved by Behörde für Justiz und Verbraucherschutz Hamburg (ORG1010, ORG946, ORG1075 and 83/19).

## Author contributions

PFK, RK, MFS, and JKS performed experiments, analyzed and interpreted data. JBE analyzed and interpreted data. SB performed experiments. VON and MAF designed the project. PFK and MAF wrote the manuscript. All authors contributed to the article and approved the submitted version.

## Supplementary material

Supplementary Table 1 Antibodies used for flow cytometry and FACS staining.

Supplementary Table 2 Differentiation factors used for the T cell differentiation assay.
