# Supplementary figures and images for: GPR52 regulates cAMP in T cells but is dispensable for encephalitogenic responses
Source: Front Immunol. 2023 Jan 24;13:1113348. doi: 10.3389/fimmu.2022.1113348 (PMC9902724; doi:10.3389/fimmu.2022.1113348)

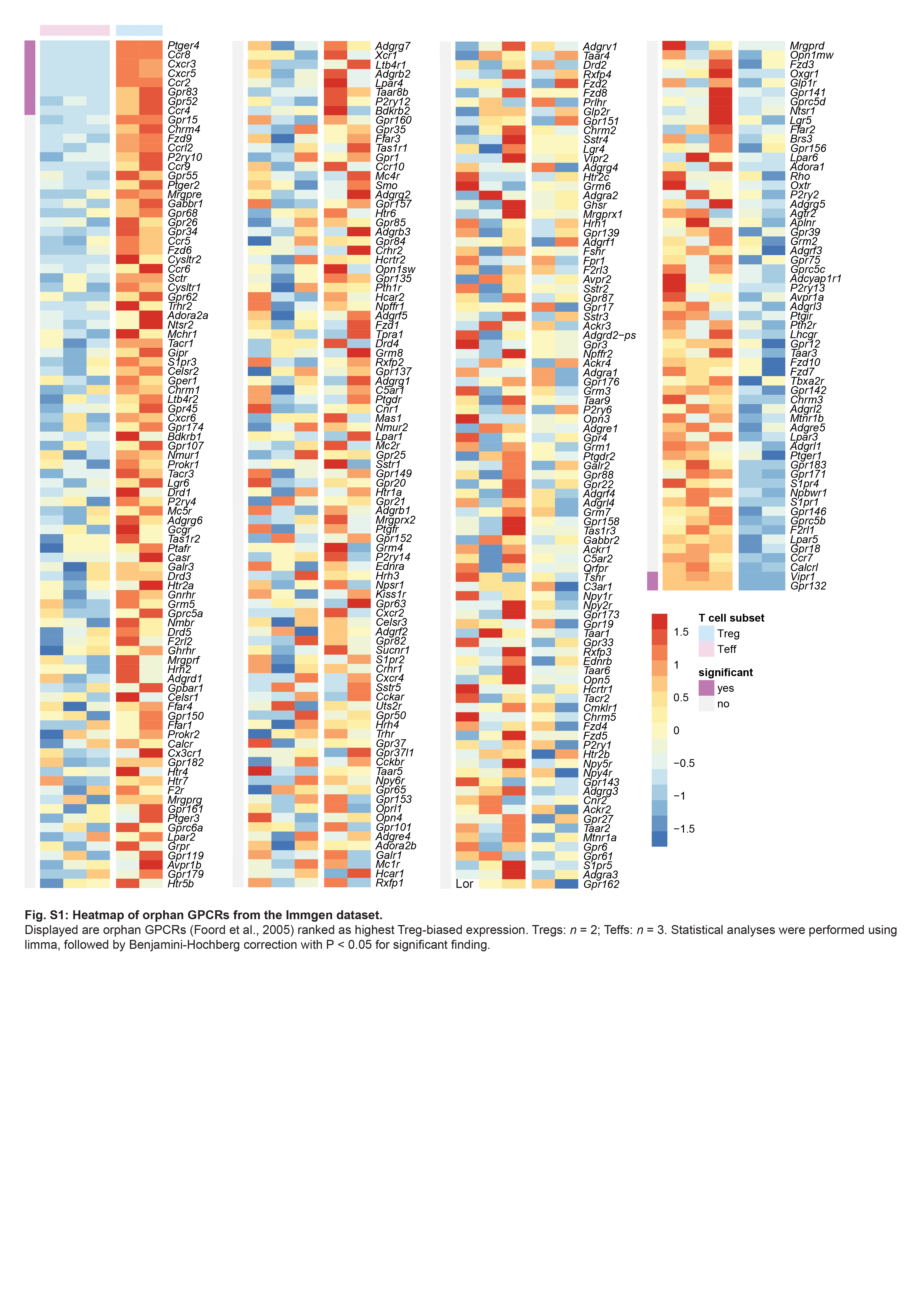

Supplement: Supplementary file 1 [file Image_1.jpeg]

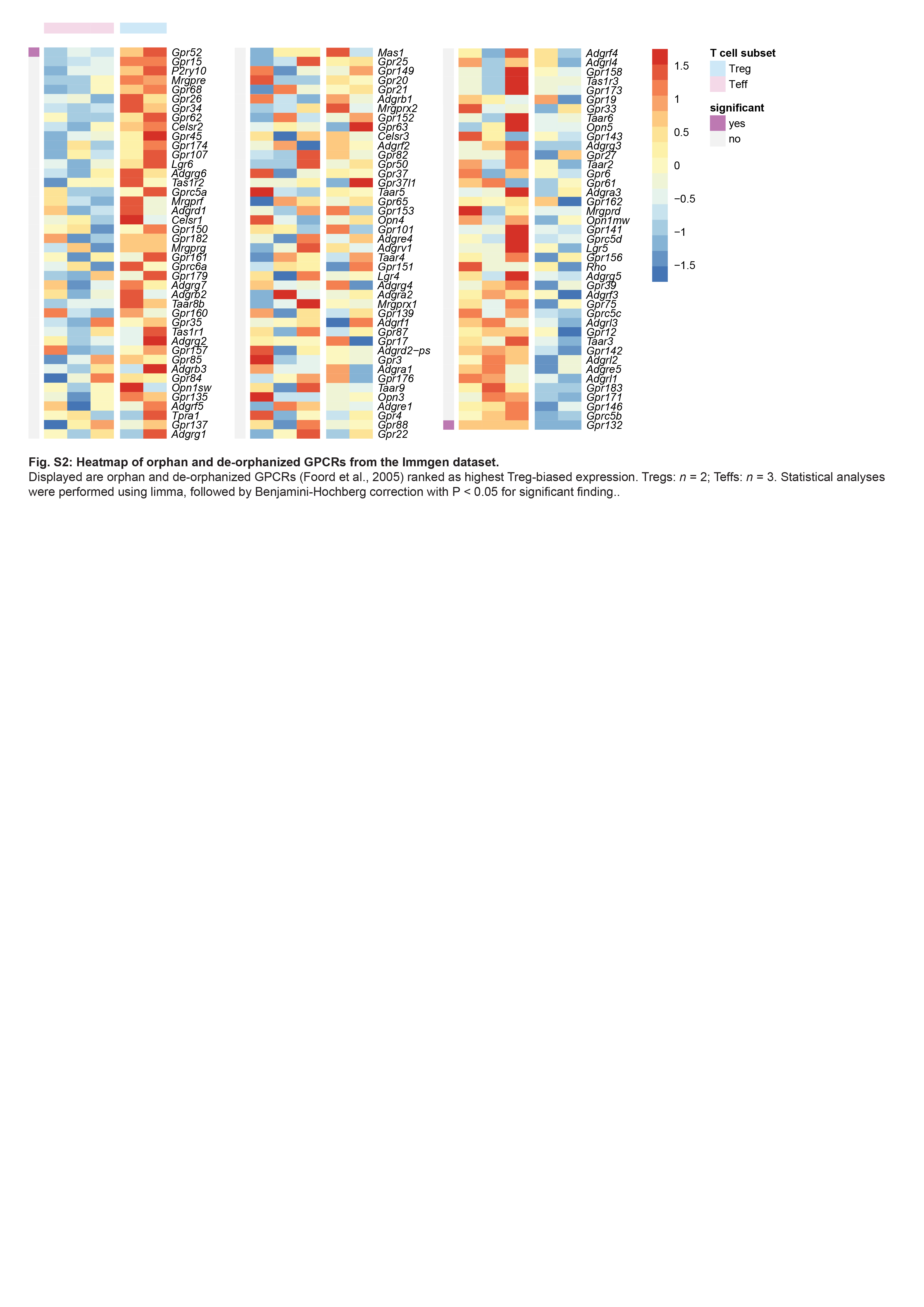

Supplement: Supplementary file 2 [file Image_2.jpeg]

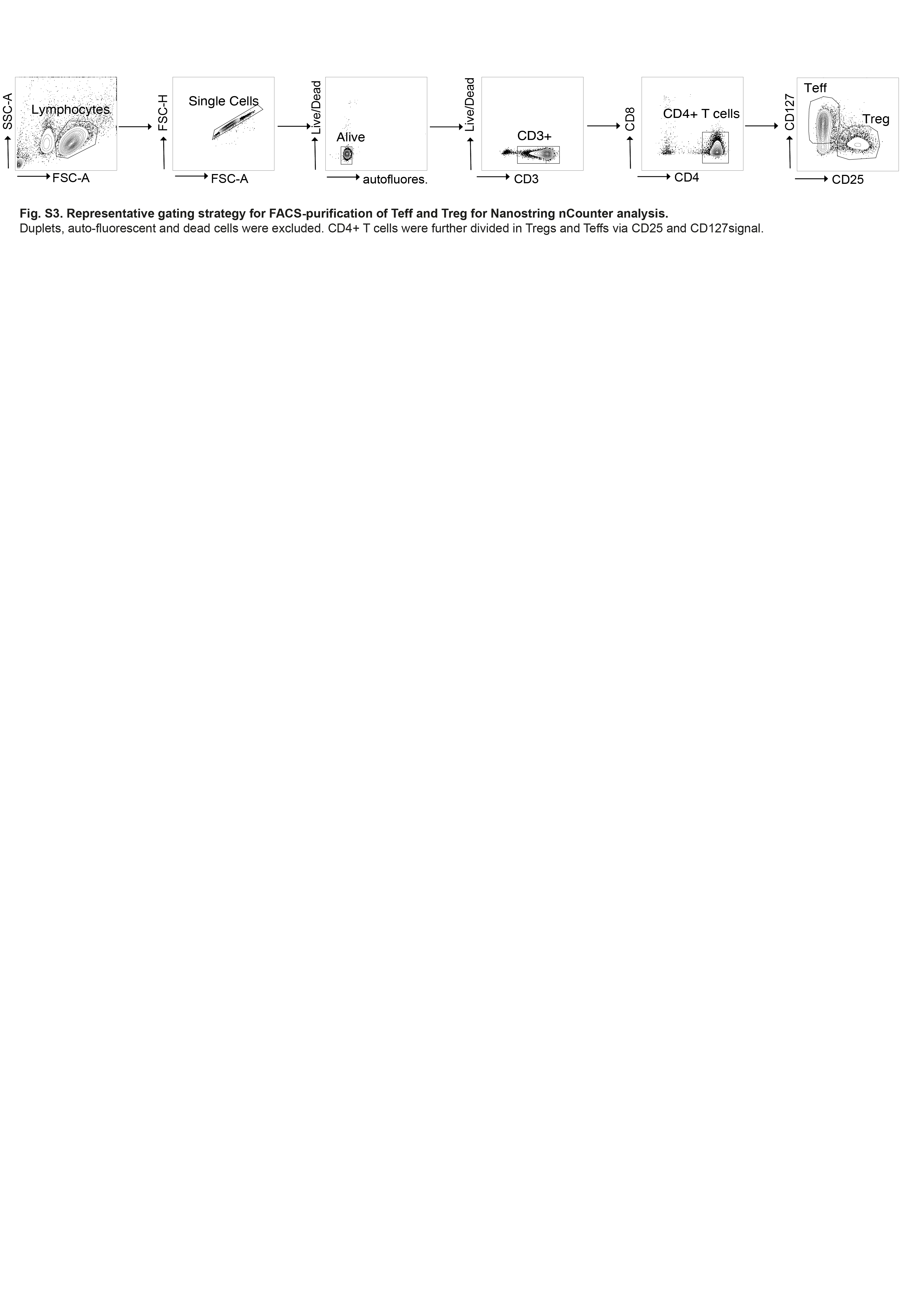

Supplement: Supplementary file 3 [file Image_3.jpeg]

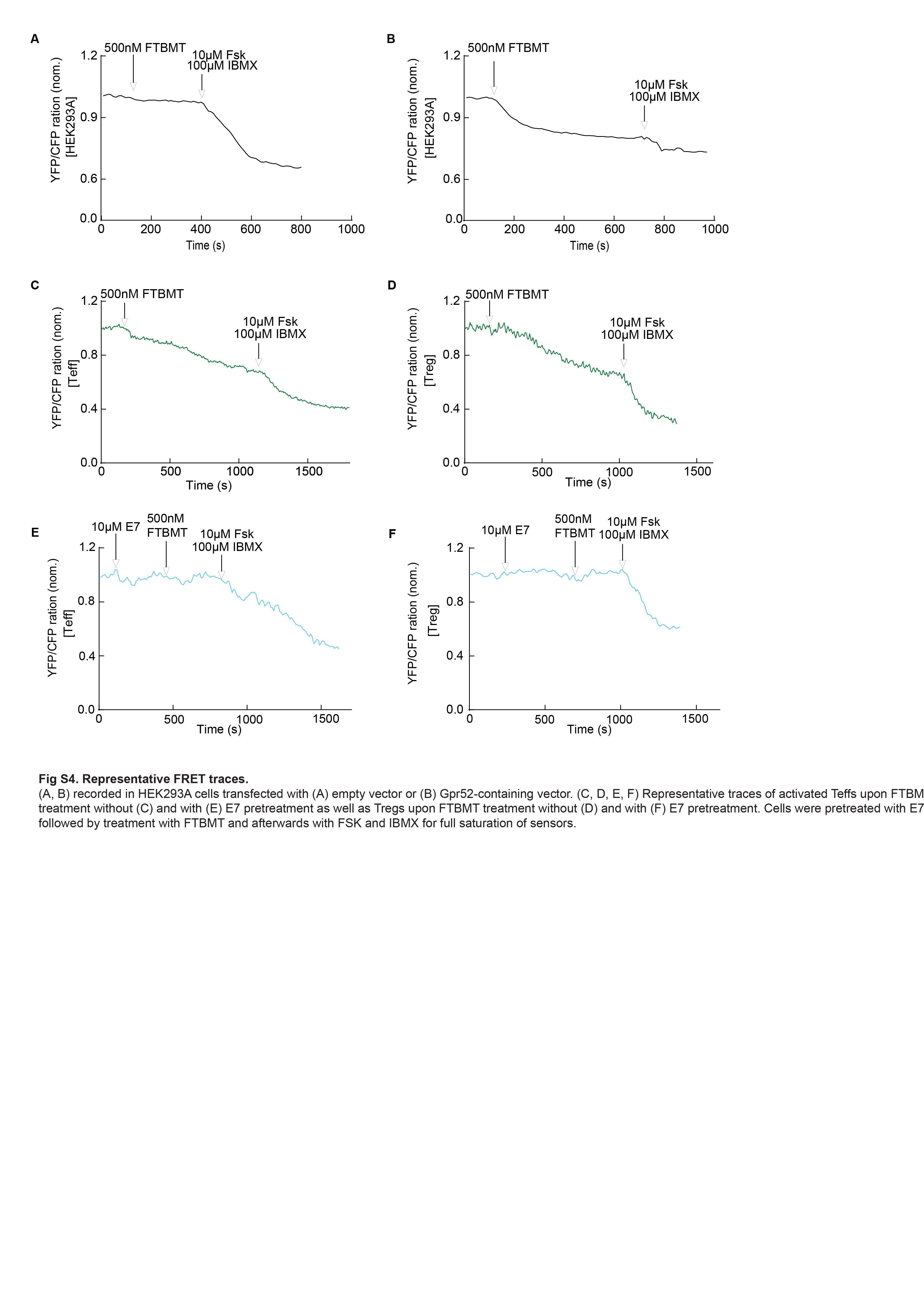

Supplement: Supplementary file 4 [file Image_4.jpeg]

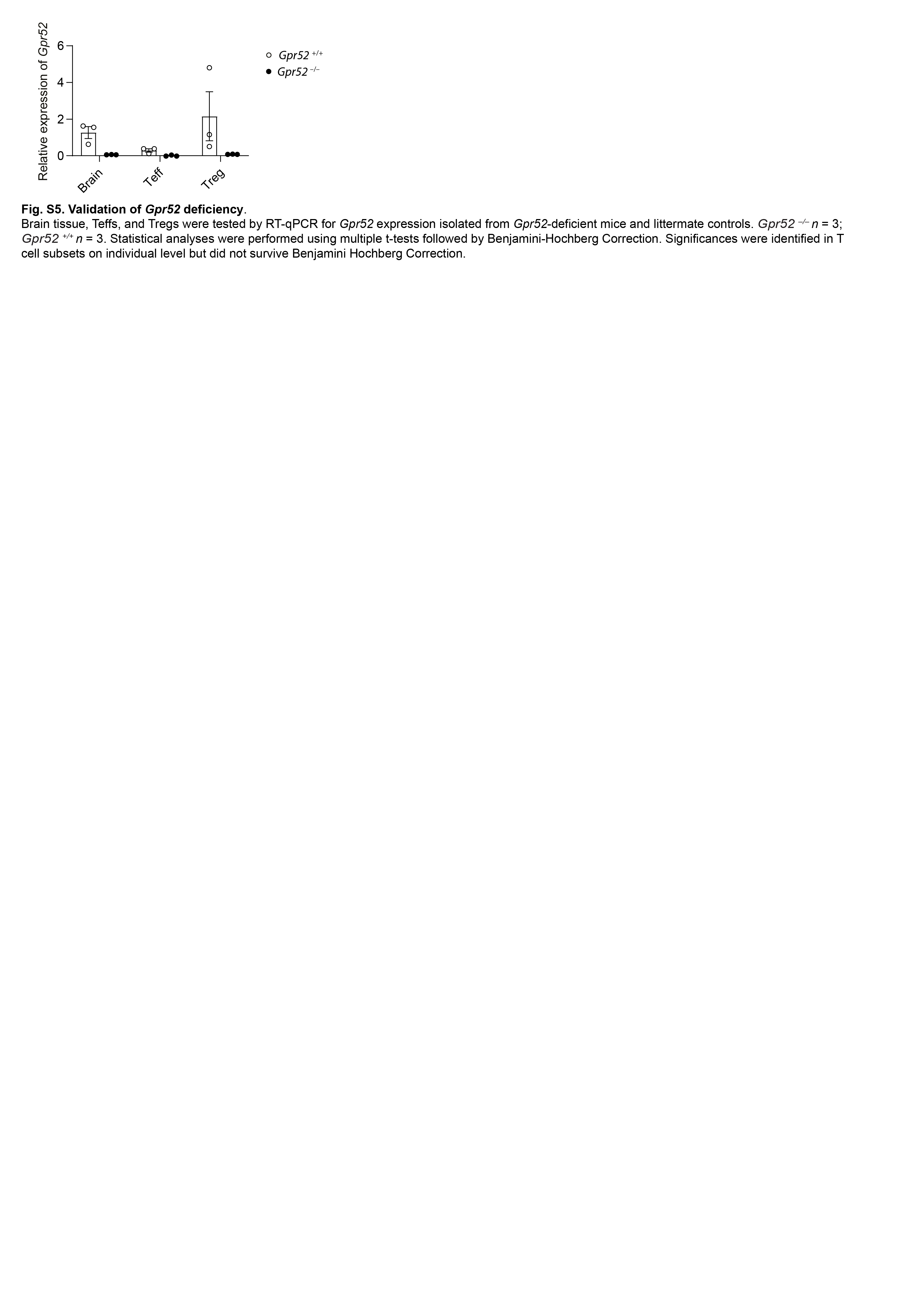

Supplement: Supplementary file 5 [file Image_5.jpeg]

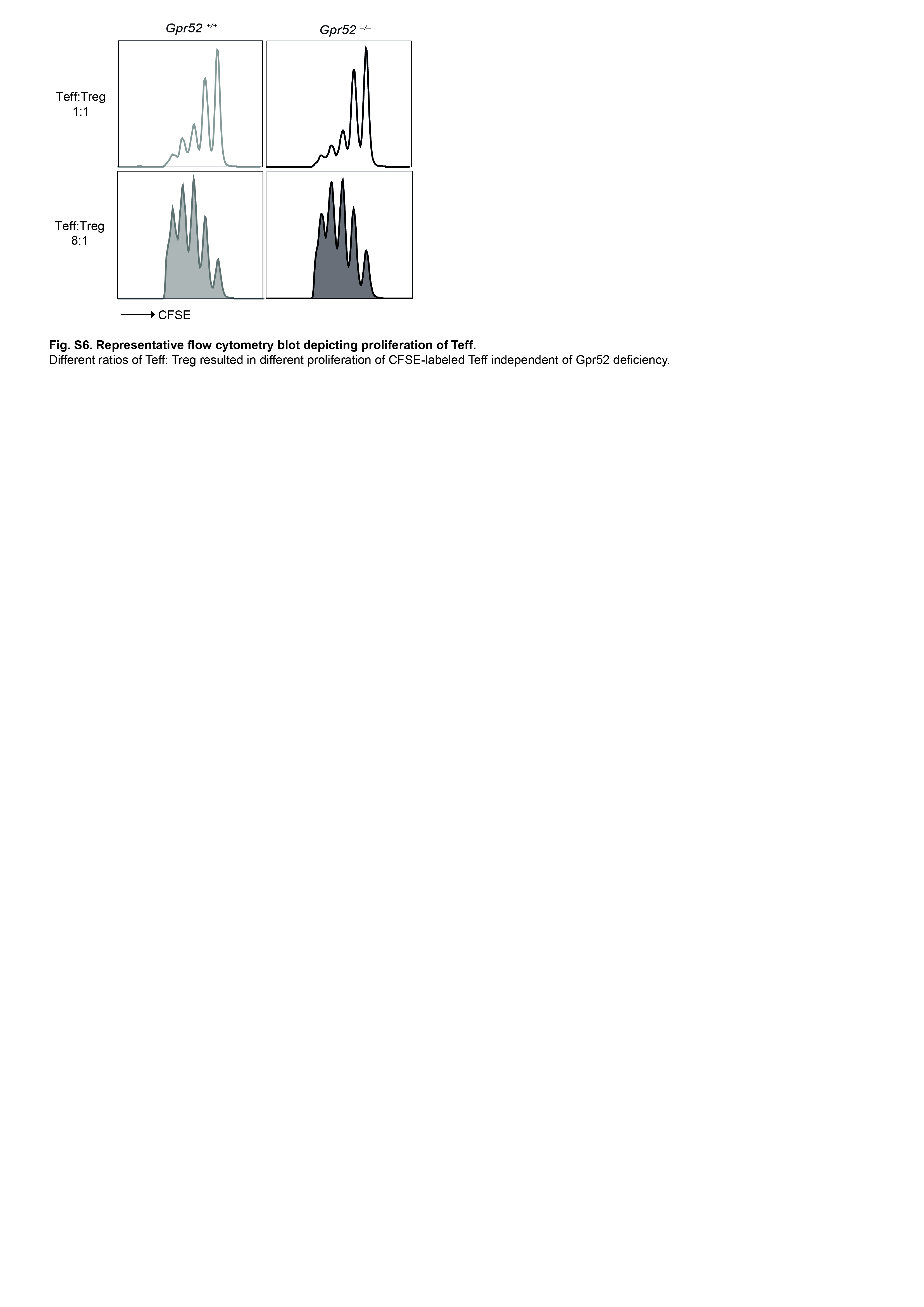

Supplement: Supplementary file 6 [file Image_6.jpeg]

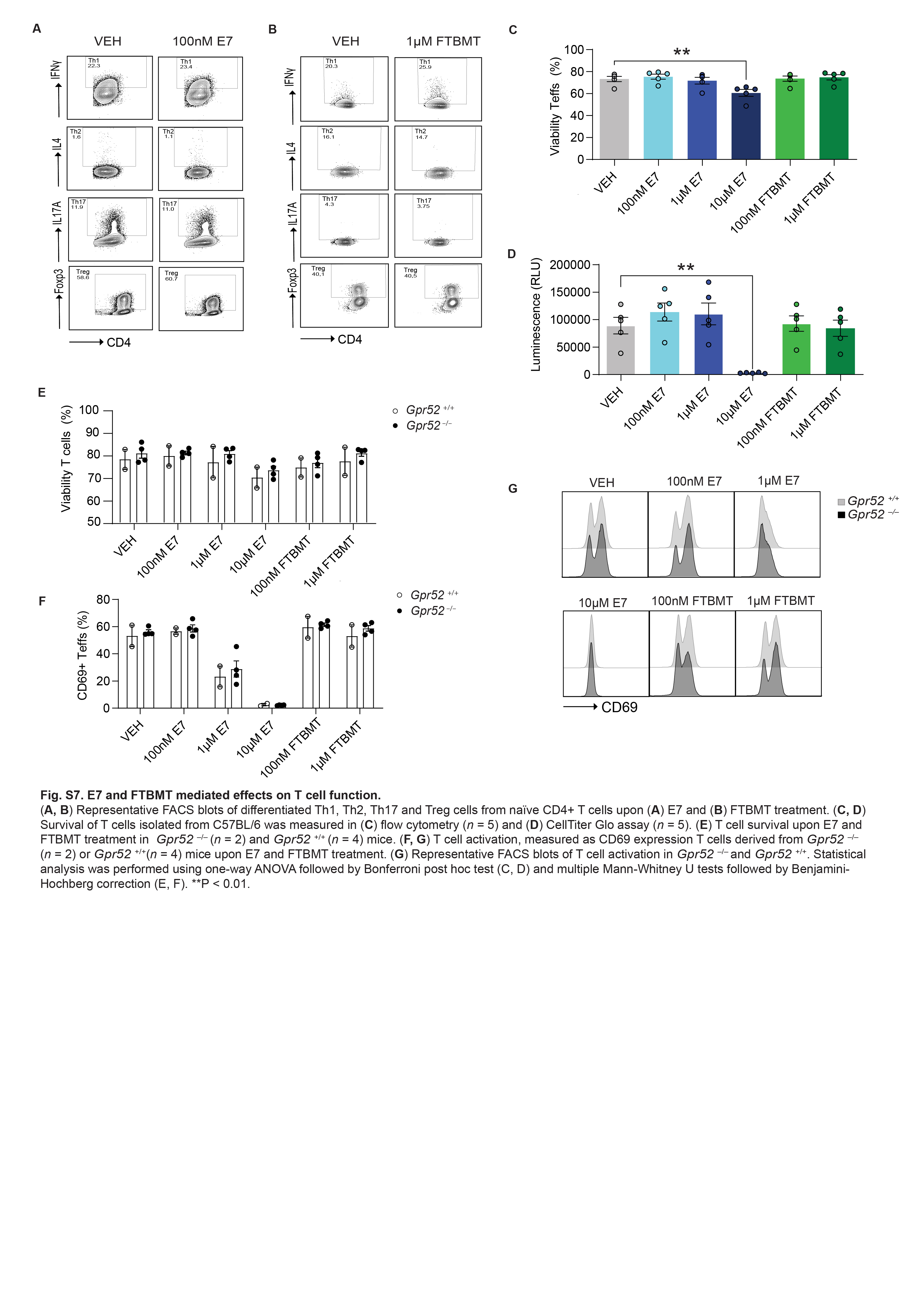

Supplement: Supplementary file 7 [file Image_7.jpeg]
